# Evaluation of the hemoglobin cutoff point for anemia in adult women residents of different altitudinal levels in Peru

**DOI:** 10.1371/journal.pone.0307502

**Published:** 2024-07-30

**Authors:** Cinthya Vásquez-Velásquez, Gustavo F. Gonzales

**Affiliations:** 1 Laboratorio de Endocrinología y Reproducción, Departamento de Ciencias Biológicas y Fisiológicas, Facultad de Ciencias e Ingeniería, Universidad Peruana Cayetano Heredia, Lima, Peru; 2 High Altitude Research Institute, Universidad Peruana Cayetano Heredia, Lima, Peru; The Ohio State University, UNITED STATES OF AMERICA

## Abstract

**Background:**

Anemia prevalence is high in countries where high proportion of the population lives at high altitude (HA) due perhaps to the unsuitability hemoglobin correction factor proposed by the WHO. The present study has been designed to evaluate a new approach to establish thresholds of hemoglobin (Hb) when defining anemia at HA.

**Materials & methods:**

Cross-sectional study evaluating 217 women aged 18 to 75 years-old, residents of 2 cities at low altitude (LA) (130 and 150 meters) and 2 at HA (3800 and 4300 meters). Hb, pulse oxygen saturation (SpO_2_), arterial oxygen content (CaO_2_), and inflammatory markers were measured. Three definitions of anemia diagnoses were used: uncorrected Hb, WHO-corrected Hb, and Silubonde’s criteria based on ferritin as a gold standard. STATA v18.0 was use for data analysis, p<0.05 indicated significant difference.

**Results:**

HA residents present higher Hb values than at LA. Likewise, the highest area under the curve (AUC) ROC (Receiver Operating Characteristic) was observed for uncorrected Hb (AUC = 0.8595; CI95% 0.858–0.86) for the diagnosis of anemia using serum ferritin as the gold standard. Anemia prevalence was higher when using WHO-corrected Hb, 27%, and Silubonde’s criteria, 41% (Hb cut-off of 11.10, 12.73, 15.80 and 16.60 g/dl for altitudes of 130, 150, 3800 and 4300 meters, respectively), than using uncorrected Hb to define anemia (7.7%). Serum Ferritin and CaO_2_ values are lower only in the group with anemia defined with uncorrected Hb than in the groups of anemia using the WHO-corrected Hb or the Silubonde´s criteria.

**Conclusions:**

The correction factor of hemoglobin for altitude of residence overestimates the prevalence of anemia in adult women. Likewise, CaO_2_ could be a potential marker to determine the transport of oxygen in LA and HA populations. Further studies in adult men are required to confirm the present findings.

## Introduction

Anemia is defined as the condition in which the oxygen-carrying capacity is insufficient to meet the body’s physiologic needs [1]. Worldwide, the amount of the population with anemia has increased from 25% in 1993–2005 to 27% in 2013 [2–4], such as the last evaluation in 2019 reports 1920 million people as having anemia. This high prevalence persists despite of strategies of intervention in most of the countries.

Hemoglobin (Hb) is an oxygen carrier but alone does not predict the oxygen transport. Arterial oxygen content (CaO_2_) is considered to correlate better with oxygen transport than hemoglobin or pulse oxygen saturation, separately. CaO_2_ is calculated from SpO_2_ and the Hb concentration in blood, recognizing that each gram of Hb can transport approximately 1.34 ml of oxygen when fully saturated [5]. Anemia is associated with a low CaO_2_ [6]_._

Prevalence of anemia is higher in countries where high proportion of the population lives at high altitudes (HA) [7]. One of the reasons behind of the high prevalence of anemia in highland populations is the adjustment of hemoglobin for altitude recommended by the World Health Organization (WHO) to define anemia [8]. This adjustment increases the prevalence of anemia, especially in moderate and high-altitude populations [9].

Different equations to determine correction factors of Hb for altitude have been reported in the literature [9–12]. These authors in basis of their own calculations propose different amounts to be subtracted from the value of Hb measured according to altitude. Several authors have questioned the utility of the adjustment of Hb by altitude [13–16].

In 2020, a new and different proposal has been reported for the altitude of Johannesburg, in South Africa (1700 meters above sea level) [17]. These authors use a new approach to determine the cutoff point of Hb to define iron deficiency anemia (IDA) using a Receiver Operating Characteristic (ROC) curve between ID (Low ferritin) and Hb (continuous variable). The Hb value with highest sensitivity and specificity to detect iron deficiency (ID) among women of reproductive age (WRA) is used as cut-off to define anemia at a particular level at 1700 m of altitude. This Hb value is used as cut-off to define anemia rather than the cut-off of Hb = 12 g/dl recommended by the WHO for WRA.

Then, it may be useful to identify if iron status (measured by serum ferritin), calculation of CaO_2_ and prevalence of anemia are different when anemia is defined using Hb not adjusted for altitude, Hb adjusted for altitude as WHO recommendation [1], and Hb adjusted according to Silubonde’s criteria [17]. Therefore, the present study aims to evaluate a new approach for anemia diagnosis comparing the three criteria mentioned above.

## Materials and methods

### Study design

This is a cross-sectional study design. The study was conducted in 217 adult women, living at different altitudes in Peru (Iquitos at 130 m, Lima at 150 m, Puno at 3800 m and Cerro de Pasco at 4340 m) in urban sectors, with availability of basic services such as electricity, water, and sewage, recruited from March 1 to July 15, 2018. Inclusion criteria: Women between 18 and 75 years-old, with more than 10 years living in the studied cities, and apparently healthy at the time of the study. Exclusion criteria: Pregnant women, with drug treatment, or with active contraceptive use [18]. Women with metabolic disease and women with systolic blood pressure ≥140 mm Hg and/or diastolic blood pressure ≥ 90 mm Hg.

### Recruitment of participants

The sampling was non-probabilistic for convenience; the research aims to establish a baseline of the variables under study.

The strategies for sample collection were announcement through the mass media; call in Work Centers, telephone companies, Ministries, universities, or urban transport companies. Then, the volunteer women were pre-recorded. Finally, evaluation and selection of volunteers, according to inclusion criteria, and exclusion criteria.

### Data collection

A venous blood sample was obtained from each woman in fasted state. Hb was measured in total blood. The rest of the blood sample was placed in a tube without additives and centrifuged at 15,000 rpm. Serum was extracted and stored at -20°C until assessments.

Hemoglobin (g/dl) was measured using the hemoglobinometer Hemocue® Hb 201+ System (Alglholm, Sweden). In the case of residents at HA, the WHO correction factor for hemoglobin was applied, and named “corrected hemoglobin”. We have corrected individual Hb values for altitude [8]. This approach is preferred to analyze hemoglobin data from populations [19]. Adjustment is the amount subtracted from observed Hb in everyone [20].

At the same time, the correction described by Silubonde et al. [17] was also applied. The two methods of Hb adjustments proposed by the WHO and Silubondi, respectively, were compared to that obtained without adjusting the Hb by altitude (uncorrected Hb).

The biomarkers of iron status and inflammation, ferritin (EIA-4292), soluble transferrin receptor (sTfR) (EIA-4256) and Interleukin-6 (EIA-4640) (DRG, Germany) were measured by ELISA following the instructions of the manufacturers.

Serum albumin was measured by spectrophotometry. Albumin is a negative acute phase protein, and as such hypoalbuminemia might represent an increased inflammatory status of the patient, [21]. Albumin production may be inhibited by pro-inflammatory mediators such as interleukin-6 (IL-6), interleukin-1 (IL-1) and tumor necrosis factor [22]. For such reason, it is considered inflammation when IL-6 is elevated, and serum albumin is lowered.

Normal ferritin was defined when values were ≥15 ng/ml and inflammatory markers (serum albumin and IL-6, levels were normal). Low ferritin values were used as <30 ng/ml when is associated to values of albumin <3.5 ng/dl and IL-6 >64 pg/ml. Soluble transferrin receptor (sTFr) was considered abnormal when ≥8.3 mg/L.

Arterial oxygen content was calculated using the following formula:

$$CaO2=\frac{SpO2\;x\;hemoglobin\;x\;1.34}{100}$$

Where SpO_2_ was expressed as percentage (%), and 1.34 ml is the amount of oxygen carried per gram of hemoglobin [23].

Pulse oxygen saturation (SpO_2_) was measured with pulse oximeter (Ohmeda Tuffsat, General Electric).

Blood pressure was measured in sitting position with an aneroid sphygmomanometer. Data were recorded as mm Hg for systolic and diastolic blood pressure (SBP and DBP). Height and weight were measured in each volunteer and body mass index (BMI) was calculated as (weight in Kg)/ (height in meters) ^2^.

### Statistical analysis

The STATA v 18.0 statistical package was used for data analysis. The values were expressed as mean ± SEM. Frequencies were presented as percentages. The ANOVA test was used to determine the difference between the means.

Sensitivity is the ability of a test to correctly identify subjects with the disease or the condition under study (true positive rate), whereas specificity is the ability of a test to correctly identify subjects without the disease or the condition under study (true negative rate).

The logistic regression analysis was also used for the construction of ROC curves to compare presence of anemia as dependent variable with serum ferritin levels.

Anemia was defined 1) when Hb uncorrected for altitude was < 12 g/dl), 2) when Hb corrected for altitude (WHO’s Adjustment) was <12 g/dl or 3) using the Silubonde’s et al. approach [17]. Silubonde’s approach allows to determine an Hb value which will be used instead of the Hb = 12 g/dl defined by the WHO as cut-off to define anemia. The Hb cut-off obtained using Silubonde’s approach will vary according to the altitude in which the studied population lives.

Data of women with inflammation were assessed using two approaches. One, without exclusion of women with inflammation but inflammatory markers (IL-6 and albumin) was controlled in the analysis. The second approach was excluding women with inflammation from the analysis.

Inflammation was defined when serum IL-6 levels was higher than 65 pg/ml [24] and serum albumin levels <3.5 g/dl [25]. p<0.05 was considered as statistically significant. For the present study only one woman had elevated IL-6 levels and lower serum albumin levels. Seven women had elevated IL-6 levels with normal values of serum albumin.

### Ethical considerations

The study was registered with SIDISI (Code: 101555), required by the Universidad Peruana Cayetano Heredia, and was reviewed by the institutional review board (Certificate: 665-20-17). The volunteers signed an informed consent, necessary to proceed with the collection of samples.

## Results

**Table 1** shows the general characteristics of the women who volunteered for the study. It shows the mean ± SEM of the age, SBP, DBP, BMI, SpO_2,_ CaO_2_ and other relevant markers (Hb, ferritin, sTfR, and inflammatory markers) in women residing in each of the cities (Lima, Iquitos, Puno, Cerro de Pasco). Corrected Hb was higher in women at 4300 m than at 150 m (p<0.05).

**Table 1:** Characteristics of women residents of the four cities (Iquitos, Lima, Puno and Cerro de Pasco) studied.

Variables		Altitude				
	Iquitos		Lima	Puno	Cerro de Pasco	
	(130 m)		(150 m)	(3800 m)	(4380 m)	
**N**	25		48	50	94	
Age (years)	38.0±2.8		37.66±1.96	35.40±1.89	38.98±1.61	
Systolic pressure (mmHg)	104.4±1.83^#^		99.15±1.68	106.86±1.30*	102.92±1.44	
Diastolic pressure (mmHg)	66.40±1.40		67.98±1.63	71.30±1.35	62.63±1.69*	
BMI (Kg/m^2^)	26.10±0.98		25.77±0.67	25.86±0.57	26.82±0.67	
SpO_2_ (%)	97.84±0.33		98.73±0.13	89.7±0.38*	87.46±0.48*	
Hb (g/dL)	12.29±0.34		13.10±0.17	15.90±0.19*	17.13±0.25*	
Adjusted Hb (g/dL)	12.29±0.34		13.10±0.17	12.77±0.18	13.16±0.25*	
Ferritin (ng/ml)	30.44±9.83		47.10±10.72	51.94±17.55	61.39±8.02	
sTfR (mg/L)	1.61±0.16		1.76±0.12	2.24±0.15	3.17±0.29*	
CaO_2_ (mL/dL)	16.09±0.45		16.22±0.64	19.12±0.24*	19.58±0.40*	
Albumins (g/dL)	4.39±0.09		4.26±0.05	4.17±0.06	4.83±0.03*^,^**	
IL-6 (pg/mL)	27.57±5.66		18.01±3.76	18.36±3.27	12.71±2.34	

Data are mean±SEM. M: meters. N = number of subjects per group. BMI: Body Mass Index. Hb: haemoglobin. SpO_2_: pulse oxygen saturation. sTfR: soluble transferrin receptor. CaO_2_: arterial oxygen content. IL-6: Interleukin 6. Adjusted Hb. Hemoglobin adjusted according to World Health Organization.

*p<0.05, with respect to Lima

#p<0.05, between sea level cities (Lima vs Iquitos)

**p<0.05, between high altitude cities (Puno vs Cerro de Pasco)

Age and body mass index were no different (p>0.05) among women in the four cities studied, while SpO_2_ was lower in women from HA than from LA. However, CaO_2_ was higher in women from HA than from LA (p<0.05).

The SBP was higher in adult women from Iquitos (130 m) than those from Lima (150 m), and in Puno than in Lima (p<0.05). DBP was lower in Cerro de Pasco compared to Lima (p<0.05). No differences were observed between other cities.

Hemoglobin concentration was higher in women residing at HA cities. Serum ferritin is similar in the 4 cities; the mean was 54.61±5.67 ng/ml. Soluble transferrin receptor (sTfR) level was similar in women residing at 130, 150 and 3800 m altitude, whereas values in women at 4340 m was higher than in the other three cities (p<0.05). Serum albumin was higher in women residing at Puno (3800 m), compared to the other 3 cities (p<0.05). However, all values were within the normal range, while IL6 values were similar in women in the 4 cities studied.

**Table 2** shows results of the sensitivity test to determine the threshold in which higher sensitivity and specificity are observed after correlating Hb (g/dl) with ID (Low serum ferritin levels) for each place of study.

**Table 2:** Cut-off points of hemoglobin obtained from ROC (Receiver Operating Characteristic) curve analysis, based on 2 diagnostic methods (ferritin<15 ng/ml, and ferritin < 30 ng/ml in cases of IL-6≥65 pg/mL, in adult women residents at 130 m, 150 m, 3800 m and 4300 m in Peru.

**130 m**	**Hb cutoff (11.10 g/dl)** *	**Hb cutoff (11.10 g/dl)** ^**#**^
Sensitivity (%)	83.30	85.70
Specificity (%)	26.30	27.80
Positive predictive value (%)	26.30	31.60
Negative predictive value (%)	83.30	83.30
**150 m**	**Hb cutoff (12.70 g/d)** *	**Hb cutoff (12.50 g/dl)** ^**#**^
Sensitivity (%)	61.10	66.70
Specificity (%)	41.40	40.70
Positive predictive value (%)	39.30	42.90
Negative predictive value (%)	63.20	64.70
**3800 m**	**Hb cutoff (16.40 g/d)** *	**Hb cutoff (15.80 g/dl)** ^**#**^
Sensitivity (%)	38.50	66.70
Specificity (%)	62.50	66.70
Positive predictive value (%)	52.60	80.00
Negative predictive value (%)	48.40	50.00
**4300 m**	**Hb cutoff (16.40 g/d)** *	**Hb cutoff (16.60 g/dl)** ^**#**^
Sensitivity (%)	58.30	52.90
Specificity (%)	35.10	40.00
Positive predictive value (%)	36.20	42.90
Negative predictive value (%)	57.10	50.00

The analysis of all altitudes and for each altitude floor was based on two criteria

* Hb Cutoff point was obtained using as marker of iron deficiency (ID) serum ferritin <15 ng/mL at each level of altitude.

^#^Hb Cutoff point was obtained using as marker of iron deficiency (ID) serum ferritin <15 ng/ml when IL6 and albumin level are within normal range, and ferritin< 30 ng/ml when IL6 was ≥65 pg/ml, at each level of altitude.

Hb cutoff is obtained as the value that maximizes combined sensitivity and specificity to detect ID.

For this assessment, an Hb threshold of 11.1 g/dl, 12.73 g/dl, 15.80 g/dl and 16,60 g/dl were obtained for altitudes of 130, 150, 3800 and 4300 m respectively when ID is defined as serum ferritin <15 ng/ml.

When ID is defined with serum ferritin levels <30 ng/ml, the Hb threshold was 12.80 g/dl, 12.30 g/dl, 14.40 g/dl, and 16.60 g/dl for altitudes of 130, 150, 3800 and 4300 m respectively. The hemoglobin values obtained from this approach were selected as the cut-off point to define anemia for each altitude studied, since it presented higher sensitivity and specificity.

**Fig 1:**
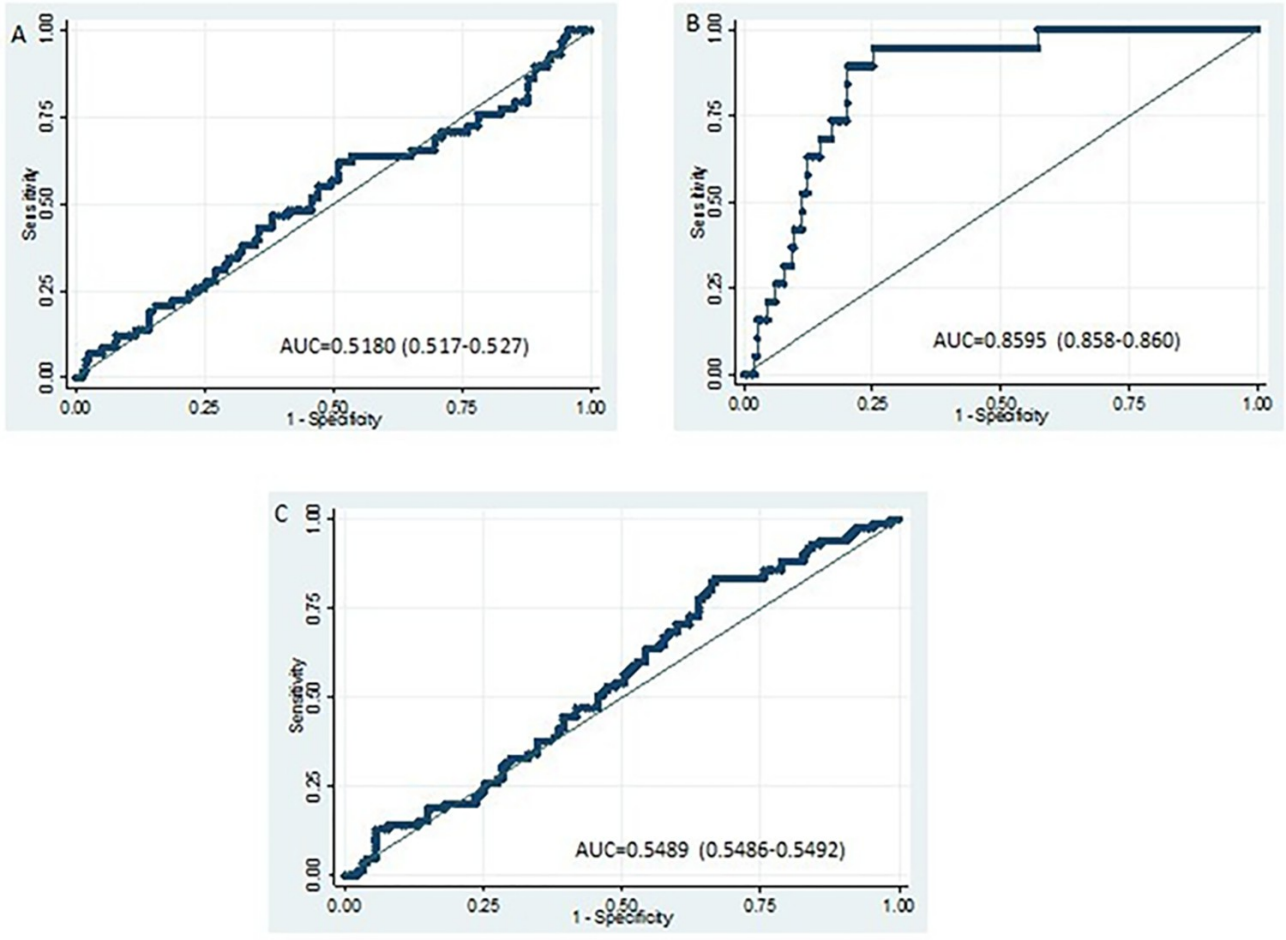
ROC curve analysis. **A.** Based on the diagnosis of anemia with corrected Hb proposed by the WHO. **B.** Based on the diagnosis with uncorrected Hb. **C.** Based on Solubonde criteria (Hb<12.4 g/dl). The areas under the curve (AUC) and the 95% confidence interval are shown in the graphs.

With these values, we have constructed ROC curves to associate anemia (dependent variable) defined by adjusted Hb for altitude (**Fig 1A**), according to the Hb unadjusted for altitude as recommended by the WHO (**Fig 1B**), and according to Silubonde’s et al. approach (**Fig 1C**). In the three curves ROC serum ferritin was used as gold standard (independent variable). The best area under the curve (AUC) ROC was observed when anemia was defined without Hb correction (AUC: 0.8595; CI 95%: 0.858–0.86) (Fig 1B). Data were obtained controlling by altitude, age and inflammatory markers (serum IL-6 and albumin levels)”.

**Fig 2:**
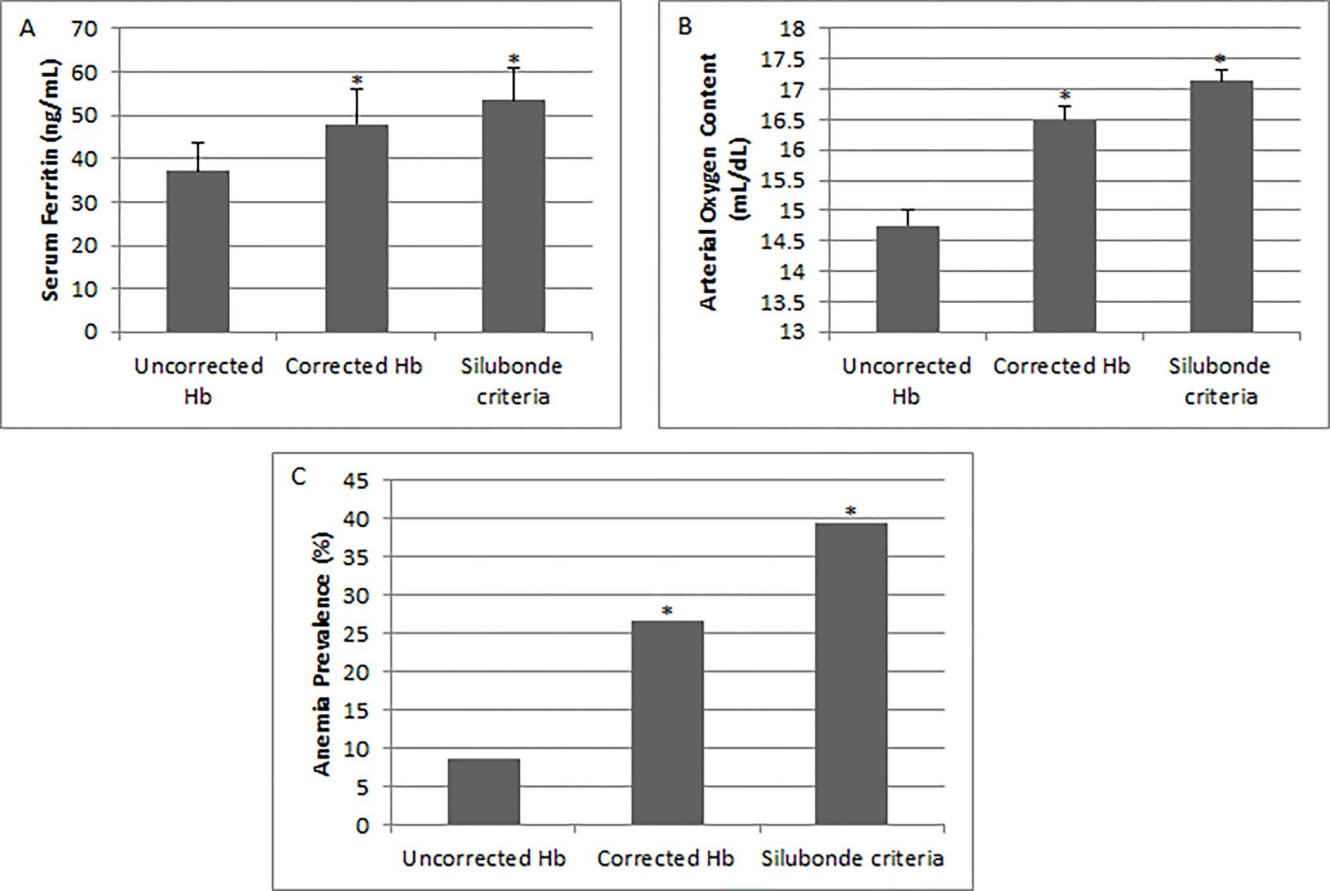
**A.** Serum ferritin in women with iron deficiency anemia (IDA). **B.** Arterial Oxygen Content in women with IDA. **C.** Prevalence of anemia diagnosed according to the criteria of uncorrected hemoglobin, corrected Hb according to WHO recommendation for altitude [1], and Hb using criteria described by Silubonde [17].

In **Fig 2A** is observed that serum ferritin is lower in women with anemia defined with uncorrected Hb than in those cases of anemia defined after Hb correction (p<0.05). Further analysis showed that serum ferritin levels were lower in women with anemia compared to normal women when Hb was uncorrected by altitude. When Hb was corrected by altitude according to the WHO recommendation, or the criteria of Silubonde et al. was used to define anemia, the values of serum ferritin were similar in women with and without anemia (p>0.05).

CaO_2_ was lower in women with anemia defined without Hb correction than in those defined with corrected Hb (WHO or with anemia defined by the Silubonde’s criteria) (**Fig 2B**).

Prevalence of anemia was 7.7% when Hb was not adjusted, and 27%, or 41% after adjustment according to the WHO or Silubonde’s criteria (using ferritin as marker) (**Fig 2C**).

## Discussion

The main function of hemoglobin is the transport of oxygen from the alveolar capillaries to the tissues. Blood oxygen content is determined by the hemoglobin concentration, and the partial pressure of oxygen in arterial blood (PaO_2_) which in turn modifies the SpO_2_ [15]. Then, the overall oxygen transport to the tissues depends on both, CaO2, and the volume of blood reaching the tissues at any given period (cardiac output) [23]. Lower CaO_2_ has been observed in situations of iron deficiency and in situations of anemia [23].

In 1958, the World Health Organization (WHO) suggested reference values to diagnose anemia based on Hb measurements. However, no revision of such parameters has been made ever since despite the mentioned report states the proposed values are determined arbitrarily [26]. As different laboratories do not agree at all with the reference values recommended by WHO, the current criteria are not being used at all around the world [27].

In 1989, the WHO recommended to adjust Hb by altitude [28]. However, this correction has been questioned by different authors [13–16, 29]. Despite of this, several authors still are proposing different ways to adjust Hb by altitude [9, 12] or to determine new cut-off to particular altitude [17].

The present study has assessed in two samples of women living at 3800 m and 4300 m the adjustment recommended by WHO, and the criteria to define cut-off of Hb by altitude [17]. Our results demonstrate that anemia defined without Hb adjustment has better AUC ROC than using the Hb adjustment by altitude according to the WHO recommendation on using this new approach.

This study adds more evidence to indicate that in Peruvian adult women the correction of Hb for altitude is inappropriate. Most of the studies criticizing the correction of Hb for altitude has been developed in children 6–59 months [7, 14–16, 20] and in pregnancy women [32]. A couple of studies in adult people were performed by Sarna et al in Ethiopia and the Tibet [13, 31] showing that WHO hemoglobin thresholds for altitude increase the prevalence of anemia in Tibetan and Ethiopian adult highlanders. However, it is still premature to generalize our finding in the Andean region. For example, it will be necessary to study the situation of adult men living in the Andean highlands.

Based on the definition of anemia described by the WHO as the deficiency of red blood cells that restrict the proper transport of oxygen to tissues to fulfill their functions, we have proposed the use of CaO_2_ as a new method to assess anemia particularly in populations living at HA. However, more studies are required.

Two other studies have shown that anemia is associated with lower CaO_2_ [6, 23]. The present study also demonstrates that CaO_2_ is lower in subjects at high altitude with anemia.

ID may be observed with and without anemia. Anemia is defined by values of Hb below a threshold. If we increase the cutoff point for Hb, as suggested by Silubonde et al. [17] trying to identify a threshold based in a ROC curve with higher sensitivity and specificity, many subjects with ID but without anemia will be erroneously classified as IDA.

Then to validate the approach presented by Silubonde et al. [17], it is needed to demonstrate that people diagnosed as having anemia really have this condition. For this, we have used the CaO_2_ and serum ferritin values.

Our results showed that women without adjustment of Hb by altitude have lower serum ferritin levels, lower CaO_2_ and lower prevalence of anemia than women diagnosed as having anemia using adjustment of Hb for altitude.

Adjustment of Hb for altitude increase the prevalence of anemia but associated with higher values of CaO_2_ and serum ferritin. These results suggest that women with normal iron store and normal CaO_2_ are being misdiagnosed as having anemia after Hb adjustment. These have been demonstrated in other studies in men living in highlands of África and Asia [13, 31]

The logic to correct Hb for altitude is that after acute exposure to hypoxia, the concentration of Hb increases but oxygen transport is reduced by the reduced arterial oxygen saturation. Then, to be valid, the correction factor of Hb for altitude must be expected to maintain CaO_2_ at similar values from sea level to altitudes above 4000 m. However, situations of low oxygen saturation and high hemoglobin concentration due to hypoxia are associated with higher CaO_2_ values at HA rather than at sea level [30]. Certainly, in our study, we have also observed higher CaO_2_ at HA than at LA suggesting that conditions for correction due to a low CaO_2_ at HA is not accomplished in residents at high altitudes. When Hb is corrected for altitude, it is expected that theoretically the concentration of Hb will be maintained at similar values at sea level and HA.

However, this will not happen with the CaO_2_ which will be higher at high altitude than at low altitude. If the CaO_2_ measurement is an indicator of oxygen transport, its assessment can replace the use of only Hb measurement for the diagnosis of anemia at HA. However, further studies would be needed to determine the utility of CaO_2_ to define anemia in different situations as those with respiratory disease who might have elevated Hb and decreased SpO_2_; or pregnant women where physiological hemodilution is observed. It is also to study this approach in adult men and in children.

The approach from the study in South Africa was to improve overall iron status when relying on a single blood test, Hb, to remain valid from a public health perspective given limited resources within community clinics. Therefore, it may be that countries have to use adjustments or to best fit their situation as the causes of ID are so diverse and are further complicated by factors like body weight, inflammation, etc.

Several studies refer that Hb should not be adjusted by altitude [7, 13, 21, 29, 31, 32]. Other studies suggest that correction underestimate anemia at lower altitudes and overestimate at higher altitudes [31]. This needs to be carefully assessed, since correction of Hb for altitude increases three to five times the prevalence of anemia. Recent studies raised the concern of treatment with iron in iron-sufficient people. Then, it is important to discuss if it is necessary or not to apply the adjustment of Hb for altitude.

Finally, our results also showed that SBP was higher in Puno (3800) than in Lima (150 m) but not with Iquitos (130 m), and DBP was higher in Lima than Cerro de Pasco but not in women from Puno (3800 m). We must be careful with the interpretation of these findings. As criteria of exclusion was women with hypertension, these exclusions could affect the mean values of SBP and DBP. As observed in other studies it is a long history of knowledge that arterial blood pressure was lower at HA than at sea level [33]. Inclusive, hypertension is more frequent at LA than at HA.

## Conclusions

Our data suggests that hemoglobin should not be corrected for altitude in the adult women in the Andean region, and that arterial oxygen content assessment could be an important tool to evaluate oxygen transport to tissues in populations living at HA since use measurements of SpO_2_ and Hb concentration simultaneously. At this time, we cannot generalize the conclusion for both sexes since more studies are required.
